# Regulation and roles of Ca^2+^ stores in human sperm

**DOI:** 10.1530/REP-15-0102

**Published:** 2015-08

**Authors:** Joao Correia, Francesco Michelangeli, Stephen Publicover

**Affiliations:** School of Biosciences, University of Birmingham, Edgbaston, Birmingham, B15 2TT, UK

## Abstract

[Ca^2^^+^]_i_ signalling is a key regulatory mechanism in sperm function. In mammalian sperm the Ca^2^^+^-permeable plasma membrane ion channel CatSper is central to [Ca^2^^+^]_i_ signalling, but there is good evidence that Ca^2^^+^ stored in intracellular organelles is also functionally important. Here we briefly review the current understanding of the diversity of Ca^2^^+^ stores and the mechanisms for the regulation of their activity. We then consider the evidence for the involvement of these stores in [Ca^2^^+^]_i_ signalling in mammalian (primarily human) sperm, the agonists that may activate these stores and their role in control of sperm function. Finally we consider the evidence that membrane Ca^2^^+^ channels and stored Ca^2^^+^ may play discrete roles in the regulation of sperm activities and propose a mechanism by which these different components of the sperm Ca^2^^+^-signalling apparatus may interact to generate complex and spatially diverse [Ca^2^^+^]_i_ signals.

## Ca^2^^+^ signalling in sperm

**Figure 1:**
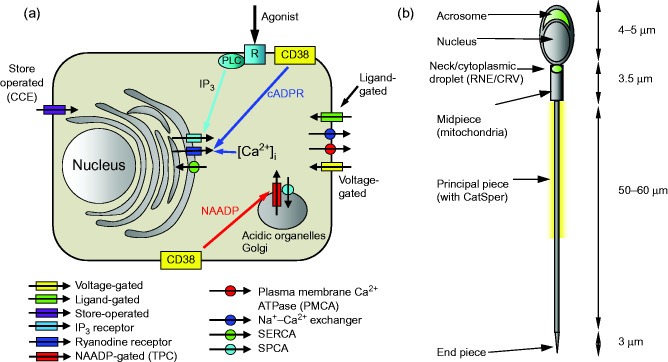
(a) Simplified diagrammatic summary of [Ca^2^^+^]_i_ signalling toolkit in a somatic cell. Ion channels are shown as rectangles with arrow indicating normal direction of Ca^2^^+^ flow (yellow, voltage-gated; green, ligand-gated; purple, store-operated; light blue, IP_3_ receptor; dark blue, ryanodine receptor; red, NAADP-gated). Pumps are shown as circles with arrows indicating normal direction of Ca^2^^+^ movement (red, PMCA'; blue, Na^+^–Ca^2^^+^ exchanger; green, SERCA; blue, SPCA). Activation of IP3 receptors by membrane receptor activation and phospholipase C is shown in light blue. Generation of cADPR and NAADP by CD38 and possibly other enzymes (leading to mobilisation of Ca^2^^+^ from intracellular stores) is shown by yellow boxes. (b) Structure of human sperm showing positions of CatSper channels (yellow shading around anterior flagellum) and Ca^2^^+^ stores in the acrosome and at the sperm neck (redundant nuclear envelope and calreticulin-containing vesicles) (shown in green).

Cellular activity is constantly regulated by environmental cues and signals from other cells. Long-term regulation of cell function is normally achieved by control of gene expression, changing the complement and levels of proteins in the cell, but rapid or short-term changes are achieved by ‘post-translational’ protein modification, such as phosphorylation, sumoylation and nitrosylation, which alter the function/activity of proteins already present. Ca^2^^+^-signalling is a key regulator of such post-translational modifications, with changes in cytoplasmic Ca^2^^+^ concentration ([Ca^2^^+^]_i_) controlling the activities of key enzymes and proteins. Large changes in [Ca^2^^+^]_i_ can be achieved ‘instantaneously’ by flux of Ca^2^^+^ into the cytoplasm from the extracellular fluid or from storage organelles (primarily the endoplasmic reticulum) within the cell (Fig. 1a). The rapidity with which [Ca^2^^+^]_i_-signals can be generated is crucial for ‘instantaneous’ cellular responses such as activation of muscle contraction and secretion of neurotransmitters that are achieved by rapid post-translational modification of protein function.

The highly condensed nucleus of sperm is transcriptionally silent (Miller *et al*. 2005, Miller & Ostermeier 2006) and translational activity is also negligible (though evidence has been presented for translation occurring at mitochondrial ribosomes; Gur & Breitbart 2008, Zhao *et al*. 2009, Chandrashekran *et al*. 2014*a*,*b*). Regulation of sperm function is therefore dependent primarily on post-translational processes. [Ca^2^^+^]_i_ signalling is pivotal to this regulation, and in mammalian sperm it plays a central role in controlling the cell's behaviour (motility type and potentially chemotaxis), the induction of acrosome reaction (AR) and the process of capacitation (Publicover *et al*. 2007, Darszon *et al*. 2007, 2011). The importance for sperm function of membrane Ca^2^^+^-channels and Ca^2^^+^-influx is well established (Darszon *et al*. 2011) but there is also good evidence for the existence and functional importance of intracellular Ca^2^^+^-storage organelles in sperm (Darszon *et al*. 2007, Publicover *et al*. 2007). Previously we reviewed the identities and functions of Ca^2^^+^ stores in sperm, focussing on the evidence for the existence of such stores, their components (pumps and channels) and their possible roles in the regulation of function in the mature sperm cell (Costello *et al*. 2009). Since then considerable progress has been made in understanding the central role of Ca^2^^+^ signalling in the regulation of mammalian and non-mammalian sperm function and the mechanisms by which sperm [Ca^2^^+^]_i_ signals are generated. In particular successful application of whole cell patch clamp technique, in human as well as mouse sperm, has revealed the central importance of Ca^2^^+^ influx through CatSper, a sperm specific, Ca^2^^+^-permeable channel in the membrane of the flagellar principal piece. Male mice null for CatSper are infertile (Ren *et al*. 2001) and their sperm show defective motility (Carlson *et al*. 2003). Here we review recent progress in understanding the diversity of mechanisms for the regulation of Ca^2^^+^ store activity and the evidence for their involvement in controlling sperm function.

## Ca^2^^+^ stores and their regulation

The importance of Ca^2^^+^ stores in generating complex Ca^2^^+^ signals in somatic cells has long been recognized. Until relatively recently the endoplasmic reticulum Ca^2^^+^ store has been the major focus for research as this was the first organelle to show controllable mobilization of Ca^2^^+^ through second messengers acting upon intracellular Ca^2^^+^ channels, as well as being able to be refilled via Ca^2^^+^ pumps. Additionally, these Ca^2^^+^ signals could also be re-modelled through the regulation of these Ca^2^^+^ transporters to generate complex spatial and temporal Ca^2^^+^ transients (Berridge *et al*. 2003). It has now become clear that many other organelles such as mitochondria, endosomes, lysosomes and Golgi complexes also contribute to the generation and propagation of these complex Ca^2^^+^ signals within cells (Michelangeli *et al*. 2005). Furthermore, novel Ca^2^^+^ transporters have also been identified within these other organelles and several have recently been identified in sperm (Costello *et al*. 2009).

### Intracellular Ca^2^^+^ channels

The major intracellular Ca^2^^+^ channels that have been identified and appear to be almost ubiquitously distributed within mammalian cells, especially on the endoplasmic reticulum, include the inositol-1,4,5-trisphosphate-(IP_3_)-sensitive Ca^2^^+^ channel (or IP_3_ receptor; IP_3_R) and the ryanodine receptor (RyR) (Michelangeli *et al*. 2005) (Fig. 1a). The IP_3_ receptor, as the name implies, is activated by the second messenger IP_3_ that is generated through the hydrolysis of phosphatidylinositol-4,5-bisphosphate. This channel has a specific IP_3_ binding site that is located towards the N-terminus of the protein (Seo *et al*. 2012) and also has a requirement for Ca^2^^+^ which acts as a co-agonist in order for the channel to open (Bezprozvanny *et al*. 1991). The activation of RyR is likely to be through a mechanism involving Ca^2^^+^ induced Ca^2^^+^ release (CICR) and by the action of the putative second messenger cyclic-adenosine diphospho-ribose (cADPR) (Ogunbayo *et al*. 2011) (Fig. 1a). cADPR is made from NAD by the action of an ADP-ribosyl cyclase enzyme such as CD38 (Cosker *et al*. 2010), although other as yet unidentified enzymes may also be involved in catalysing this reaction (Guse 2015). It is as yet unclear whether, unlike the IP_3_R, cADPR binds directly to RyR or whether it binds to accessory proteins such as calmodulin or FK506-binding protein, that then interact with the RyR (Guse 2015).

Another metabolite of NAD which is believed to have Ca^2^^+^ mobilizing ability is nicotinic acid adenine dinucleotide phosphate (NAADP; Genazzani *et al*. 1997). NAADP is made from NADP through the action of either CD38 acting as a base-exchanger, swapping the nicotinamide group for nicotinic acid or via an unidentified NADP-deaminase (Guse 2015). NAADP is believed specifically to mobilize Ca^2^^+^ from acidic stores such as lysosomes (Churchill *et al*. 2002, Menteyne *et al*. 2006), which can then induce CICR at RyRs and IP_3_Rs in mammalian cells (Cancela *et al*. 1999; Fig. 1a). Results initially presented by Calcraft *et al*. (2009), indicated that NAADP specifically activates Ca^2^^+^-specific two-pore channels (TPC) within the acidic organelles, these channels being first described in plants (Peiter *et al*. 2005). However, in kinetic studies there is a prominent lag between addition of NAADP and Ca^2^^+^ mobilization (Genazzani *et al*. 1997). Combined with the observation that photo-affinity labelling with azido-NAADP (Lin-Moshier *et al*. 2012) showed labelling of only low molecular weight proteins, not consistent with TPCs, it suggests that NAADP might function by binding to accessory proteins rather than directly to the channel. Recently there has been considerable controversy as to whether the NAADP-sensitive Ca^2^^+^ channel is a TPC (Morgan & Galione 2014). Data from two studies (Wang *et al*. 2012, Cang *et al*. 2013) suggested that TPCs are in fact Na^+^-specific channels with very low Ca^2^^+^ selectivity that are activated by phosphoinositide lipids and modulated by mTOR, but not by NAADP. However, recently published work with cells from mice null for TPC1 and TPC2 provided strong evidence that TPCs are similarly permeable to Ca^2^^+^ and Na^+^ and are NAADP-gated through binding to an accessory protein (Ruas *et al*. 2015).

Numerous kinases have been shown to modulate the activity of both the IP_3_Rs and RyRs, including several ubiquitous ser/thr kinases such as PKA, PKG and CaMKII (Yule *et al*. 2010, Camors & Valdivia 2014). Indeed, some of these kinases such as PKA appear to have both stimulatory and inhibitory effects on the IP_3_R, dependent upon isoform subtype and the presence of multiple kinase-dependent phosphorylation sites on the same receptor (Dyer *et al*. 2003). Less ubiquitous ser/thr kinases such as Akt and polo kinases as well as tyrosine kinases such as fyn kinase have also been shown to affect these channels (Yule *et al*. 2010, Camors & Valdivia 2014).

Both the RyRs and the IP_3_Rs are modulated by changes in their oxidation states caused by reactive oxygen species (ROS) and reactive nitrogen species (RNS), and this occurs mainly through modification of specific cysteine (cys) amino acid residues. Oxidation of these cys residues in RyRs occurs both by S-glutathionylation as well as S-nitrosylation by the second messenger nitric oxide (NO; Csordas & Hajnoczky 2009) and promotes the activity of the channel by enhancing RyR subunit interactions and also by reducing the efficacy of inhibitory modulators (Hamilton & Reid 2000). In IP_3_Rs the effects of oxidative stress are complex: low levels of cys oxidation caused by low concentrations of thimerosal (a cys–modifying mercuric compound) and naturally generated ROS cause sensitization of this channel, while higher concentrations of thimerosal inhibit channel activity (Missiaen *et al*. 1991, Sayers *et al*. 1993). Currently, however, there is little evidence that NO can affect the activity of the IP_3_Rs.

### Intracellular Ca^2^^+^ pumps

The major transporter involved in refilling Ca^2^^+^ stores within the endoplasmic reticulum is the sarcoplasmic/endoplasmic reticulum Ca^2^^+^ ATPase (SERCA; Fig. 1a), and these pumps occur abundantly in all somatic cells. Their role is to pump Ca^2^^+^ back into the storage organelles to help terminate Ca^2^^+^ signals (Michelangeli *et al*. 2005, Michelangeli & East 2011). There are three isoforms of this Ca^2^^+^ ATPase, each encoded by a different gene and each isoform can exist in a variety of spliced variants that differ in size and regulatory properties (Michelangeli & East 2011). SERCA1 is mainly confined to skeletal muscle, while SERCA2 is widely distributed in most other tissues and organs and type 3 has a limited expression. Another related Ca^2^^+^ ATPase that is also found ubiquitously within somatic cells is the secretory pathway Ca^2^^+^ ATPase (SPCA), which is localized to the Golgi apparatus (Wootton *et al*. 2004). SPCA exists in two isoforms with the expression of type 1 being far more widespread than type 2, which appears to be mainly located within glandular tissues (Vanoevelen *et al*. 2005). Recently there has been evidence to suggest that SPCA2 can interact with and regulate the plasma membrane located ORAI Ca^2^^+^ channels that are implicated in store-operated Ca^2^^+^ entry (Feng *et al*. 2010), which may indicate a dual function for this Ca^2^^+^ ATPase in cells that express it.

There is currently some debate as to which type of intracellular Ca^2^^+^ ATPase is expressed in mature sperm. We have highlighted that SPCA1 is present in human sperm, where it appears to be mainly localized to the neck region of the cell where the redundant nuclear envelope (RNE) and calreticulin-containing vesicles are situated (Harper *et al*. 2005). This study also found no evidence for expression of SERCA in human sperm as no cross-reactivity was observed with a pan-isoform SERCA antibody and no effects on [Ca^2^^+^]_i_ were observed with specific but saturating concentrations of the SERCA-inhibitor thapsigargin. However, a more recent study (Lawson *et al*. 2007) detected SERCA2, mainly localized to the acrosome and mid-piece, using a SERCA2-specific antibody.

Unlike the intracellular Ca^2^^+^ channels, there is no strong evidence to suggest that either SERCA or SPCA can be directly phosphorylated and regulated by protein kinases, although some Ca^2^^+^ ATPase modulatory proteins like phospholamban (that is found almost exclusively in heart) are regulated through phosphorylation by PKA, PKG and CamKII (Colyer 1998). There is considerable evidence indicating that oxidative stress can modulate SERCA activity (although no studies have yet been undertaken on SPCA). Again a number of critical cys residues such as cys674 can be S-glutathionylated to cause an increase in SERCA pump activity (Adachi *et al*. 2004). Modifications of other cys residues on the Ca^2^^+^ ATPase, however, can have inhibitory effects (Sayers *et al*. 1993, Sharov *et al*. 2006, Csordas & Hajnoczky 2009).

## Ca^2^^+^ stores, mechanisms for store mobilisation and store-operated Ca^2^^+^ channels in sperm

During the later stages of their development spermatozoa shed much of their cytoplasm including intracellular organelles. Thus mammalian sperm contain no organised endoplasmic reticulum. However, studies on the expression of Ca^2^^+^ store components and on the generation [Ca^2^^+^]_i_ signals suggest that the remaining intracellular organelles function as Ca^2^^+^-stores and play a significant role in the regulation of cellular function (Costello *et al*. 2009). In particular, the acrosomal vesicle at the apex of the head and the collection of vesicular membranous structures that occur at the sperm neck and anterior midpiece (including the cytoplasmic droplet of human sperm) appear to be functionally important Ca^2^^+^-stores (Fig. 1b; shown in green). At both these locations IP_3_Rs have been detected in human and in bovine sperm by immuno-staining (Dragileva *et al*. 1999, Kuroda *et al*. 1999, Ho & Suarez 2001, 2003, Naaby-Hansen *et al*. 2001). RyRs have also been detected in human and rodent sperm (Trevino *et al*. 1998, Lefievre *et al*. 2007). Staining of human sperm with anti-RyR1, anti-RyR2, pan-RyR and BODIPY-FLX ryanodine is localised primarily to the neck region, though some acrosomal staining was also observed (Harper *et al*. 2004, Lefievre *et al*. 2007, Park *et al*. 2011). In contrast, other authors (Ho & Suarez 2001) have reported no staining of bovine sperm with BODIPY-FLX ryanodine (see Costello *et al*. (2009) for further discussion). Thus mobilisation of stored Ca^2^^+^ in mammalian sperm may occur in response to generation of IP_3_ by activity of phospholipase C and by CICR at IP_3_Rs or RyRs. These processes can be sensitised by effects such as oxidative stress and S-nitrosylation (see ‘Ca^2^^+^ stores and their regulation’). For instance, exposure of human sperm to NO at levels equivalent to those produced by explants of reproductive tract lining mobilises stored Ca^2^^+^ and modifies flagellar activity (Lefievre *et al*. 2007, Machado-Oliveira *et al*. 2008).

In addition to generation of IP_3_ in sperm, there is evidence that other Ca^2^^+^ mobilising messengers (NAADP and cADPR) are synthesised in sperm and/or produced in response to stimulation. Sea urchin sperm contain significant levels of both cADPR and NAADP, which may contribute to oocyte activation (Chini *et al*. 1997, Billington *et al*. 2002). Human sperm have been shown to contain cADPR at micromolar concentrations but NAADP was not detected (Billington *et al*. 2006). Interestingly, this study also demonstrated synthesis of cADPR by human sperm but the ecto-enzyme CD38 (an enzyme present on mammalian cells that synthesises both cADPR and NAADP; see ‘Ca^2^^+^ stores and their regulation’) could not be detected by western blotting. In contrast, Park *et al*. (2011), reported detection of CD38 in human sperm after co-incubation with prostasomes (prostate-derived membrane vesicles; see below). Furthermore, the presence of a novel NAADP synthase, which lacks the cyclase activity of CD38, has been described both in sea urchin (Vasudevan *et al*. 2008) and human sperm (Sanchez-Tusie *et al*. 2014). In sea urchin sperm this enzyme is strongly Ca^2^^+^-regulated and most active at acid pH whereas the human enzyme shows only weak Ca^2^^+^-regulation and activity is maximal at pH 7–8 (Vasudevan *et al*. 2008, Sanchez-Tusie *et al*. 2014).

Recent findings have supported the idea that NAADP is functional in human sperm. Sanchez-Tusie *et al*. (2014) investigated the effects of cell-permeant (AM-ester) derivatives of NAAPD and cADPR. No effects were observed with cADPR, consistent with previous pharmacological investigation by Billington *et al*. (2006), but NAADP caused elevation of [Ca^2^^+^]_i_ both in cells incubated under standard conditions and also when [Ca^2^^+^]_o_ was buffered to 100 nM, conditions under which Ca^2^^+^ influx is negligible and [Ca^2^^+^]_i_ signalling depends solely on mobilisation of stored Ca^2^^+^. Staining of NAADP receptors using the fluorescent NAADP receptor ligand Ned-19 and identification of acidic organelles using lysotracker highlighted both an anterior store (potentially the acrosome) and a store at the sperm neck (Fig. 1b). Consistent with these findings, Arndt *et al*. (2014), studying AR (see below), provided evidence for involvement in this process of NAADP and TPCs, which have been proposed to be the NAADP receptor/Ca^2^^+^ channel of acidic Ca^2^^+^ storage organelles (Calcraft *et al*. 2009; Fig 1a; see ‘Ca^2^^+^ stores and their regulation’).

Park *et al*. (2011) investigated the incorporation into human sperm of proteins from prostasomes (prostate-derived vesicles which are normally added to sperm during ejaculation) and their effects on [Ca^2^^+^]_i_ signalling. They concluded that CatSper channel proteins were present in the differentiated sperm, but other Ca^2^^+^ signalling ‘tools’ including RyRs and CD38 were added to the freshly-ejaculated sperm upon mixing with prostasomes, by fusion with the membrane of the midpiece. They also examined the effects of stimulation with progesterone on [Ca^2^^+^]_i_ and motility of sperm exposed to prostasomes and sperm that had been rapidly removed from semen to minimise mixing with prostasomes. Their data suggest that the generation of sustained [Ca^2^^+^]_i_ signals (such as the second component of the biphasic progesterone-induced [Ca^2^^+^]_i_ signal) and consequent effects on motility may depend, at least partly, upon generation of cADPR by prostasome-derived enzymes. Interestingly, CD38-null mice proved to be fertile, but analysis showed that 20% of normal ADPR cyclase activity remained in prostasomes from these animals, indicating the presence of a non-CD38 ADPR-cyclase, potentially that described by Sanchez-Tusie *et al*. (2014). Thus both NAADP and cADPR are potentially synthesised by sperm and involved in regulation of sperm Ca^2^^+^ store activity but their roles are not yet clear.

In somatic cells mobilisation of stored Ca^2^^+^ induces secondary Ca^2^^+^ influx through channels at the cell membrane (store-operated channels, SOCs) by the process of capacitative Ca^2^^+^ entry (CCE) (Fig. 1a). CCE both prolongs Ca^2^^+^ signals that are induced by store mobilisation and provides Ca^2^^+^ for re-charging of the storage organelles. Recently great progress has been made in elucidating the key players and mechanisms in this process. Stromal interaction molecule (STIM) has been identified as the sensor molecule present in the membrane of the Ca^2^^+^ store. The intraluminal part of STIM includes a Ca^2^^+^-binding EF hand that detects depletion of stored Ca^2^^+^. STIM then redistributes, moving to a position adjacent to the plasma membrane where it activates channel proteins (ORAI and possibly members of the TRPC (transient receptor potential canonical) family (Cahalan 2009)). [Ca^2^^+^]_i_ signals in human and other mammalian sperm induced by agonists and by treatments designed to mobilise stored-Ca^2^^+^ show characteristics consistent with the occurrence of CCE (Blackmore 1993, Dragileva *et al*. 1999, O'Toole *et al*. 2000, Park *et al*. 2011, Lefievre *et al*. 2012). STIM1, ORAI and TRPC proteins have been detected in human sperm (Castellano *et al*. 2003, Darszon *et al*. 2012, Lefievre *et al*. 2012), STIM1 being localised primarily to the neck region/midpiece and the acrosome where Ca^2^^+^ stores are present (Lefievre *et al*. 2012). To date the application of whole-cell patch clamp has not provided evidence for the occurrence of CCE in human sperm (Lefievre *et al*. 2012) so these findings must be interpreted cautiously, but [Ca^2^^+^]_i_ signals generated by mobilisation of Ca^2^^+^ stores in sperm may be amplified by activation of CCE. Induction of CCE in somatic cells can have a latency of tens of seconds due to the need for STIM to migrate to the peripheral portions of the endoplasmic reticulum where it can interact with SOC proteins (Luik *et al*. 2006, Wu *et al*. 2006), but in sperm the storage organelles are close to the plasma membrane and STIM proteins are localised here, such that CCE could be near ‘instantaneous’. Pre-treatment of human sperm with low concentrations of 2-aminoethoxydiphenyl borate, which potentiates CCE by promoting the interaction of STIM with SOCs (Navarro-Borelly *et al*. 2008, Wang *et al*. 2009, Yamashita *et al*. 2011) significantly enhanced the amplitude of the progesterone-induced Ca^2^^+^ transient at the sperm neck (where secondary release of stored Ca^2^^+^ may occur; Fig. 1b; see ‘Model for interaction of CatSper channels and Ca^2^^+^-stores’) but did not affect the response in the flagellum, where progesterone activates CatSper channels (Fig. 1b), or the kinetics of the signal at either location (Lefievre *et al*. 2012). Conversely, when sperm were pre-treated with a cell-penetrating peptide that mimics part of the key SOAR region of STIM1 (potentially preventing auto-inhibitory folding of STIM upon store-refilling) there was a marked prolongation of the progesterone-induced [Ca^2^^+^]_i_ transient in a subset of cells (Morris *et al*. 2015).

## Mobilisation of sperm Ca^2^^+^ stores by agonists

In the majority of somatic cells mobilisation of stored Ca^2^^+^ occurs upon agonist-induced synthesis of Ca^2^^+^ mobilising intracellular messengers. Thus agonist-induced synthesis of inositol trisphosphate, cADPR and NAADP can lead to rapid release of stored Ca^2^^+^ and generation of local, global and complex spatio-temporal signals (Fig. 1a). Is there evidence that such processes occur and are functionally significant in responses to agonist stimulation of sperm?

The best-characterised agonist-induced [Ca^2^^+^]_i_ signals in sperm are responses to solubilised zona pellucida/zona proteins in mouse cells and progesterone in human. Application of patch clamp has clearly shown that the primary action of progesterone in human sperm is to activate CatSper channels, leading to Ca^2^^+^-influx (Lishko *et al*. 2011, Strunker *et al*. 2011). Strunker *et al*. (2011) investigated the [Ca^2^^+^]_o_ dependence of progesterone-induced [Ca^2^^+^]_i_ signals in rapid-mixing experiments on human sperm and reported that buffering of [Ca^2^^+^]_o_ to ≤100 nM abolished the response (though see Espino *et al*. (2009)), suggesting that any mobilisation of stored Ca^2^^+^ is a secondary response. Synthesis of IP_3_ is reported to occur downstream of progesterone-induced Ca^2^^+^ influx (Thomas & Meizel 1989), an important observation that should be pursued. Stimulation of mouse sperm with zona proteins induces AR, which requires elevation of [Ca^2^^+^]_i_ in the sperm head (Florman *et al*. 2008) and is dependent on mobilisation of Ca^2^^+^ from the acrosomal store (De Blas *et al*. 2002; see below). The nature of the Ca^2^^+^ influx following stimulation is not clear and several channels may be involved (Florman *et al*. 2008, Xia & Ren 2009, Cohen *et al*. 2014), but Ca^2^^+^ signals are sensitive to inhibition of G-protein signalling (using pertussis toxin) and inhibition of PLC (Florman *et al*. 2008, Ren & Xia 2010). Furthermore, in sperm from mice null for PLCδ4 (in which males' fertility is severely impaired) the [Ca^2^^+^]_i_ response is reduced and zona-induced AR does not occur (Fukami *et al*. 2001, 2003). Thus conventional IP_3_-induced mobilisation of stored Ca^2^^+^ is apparently central to this essential aspect of mammalian sperm physiology.

Evidence for the existence of other store-mobilising agonists is largely preliminary, but there are a number of candidates, of which the best-studied is vitamin D (Blomberg Jensen 2014). Human sperm have been shown to express vitamin D receptor (VDR; Aquila *et al*. 2009, Blomberg Jensen *et al*. 2010, 2011), the enzymes CYP2R1 and CYP27B (which produce the active compound (1,25(OH)_2_D_3_) cholecalciferol) and the inactivating enzyme CYP24A1 (Blomberg Jensen *et al*. 2010, 2011). All are expressed in the neck region of the sperm and staining of cells for VDR and CYP24A1 shows a strong association. In sub-fertile patients the proportion of cells expressing CYP24A1 varies greatly and is significantly correlated with semen quality (sperm count, concentration, morphology and motility; Blomberg Jensen *et al*. 2011, 2012). Stimulation of human sperm with 1,25(OH)_2_D_3_ (100 pM–1 μM) induced a [Ca^2^^+^]_i_ response, including a transient and plateau, that was blocked by pre-treatment with the non-genomic VDR antagonist 1β,25(OH)_2_D_3_ but was insensitive to the nuclear VDR antagonist ZK159222 (Blomberg Jensen *et al*. 2011). This effect was greatly reduced by pre-treatment with the phospholipase C inhibitor U73122 (2 μM) but was also inhibited by incubation in EGTA-buffered medium for up to 20 min prior to stimulation. Both motility and AR were significantly increased upon stimulation with 1,25(OH)_2_D_3_ (Blomberg Jensen *et al*. 2011).

Kisspeptin, a peptide agonist of the G-protein coupled receptor GPR54/KISS1R, has also been shown to cause sustained, dose-dependent elevation of [Ca^2^^+^]_i_ in human and in mouse sperm (Pinto *et al*. 2012, Hsu *et al*. 2014). In neurons binding of kisspeptin to its receptor activates PLC and results in generation of IP_3_ and diacyglycerol, leading to mobilisation of stored Ca^2^^+^ and also depolarisation (Liu *et al*. 2008, Pielecka-Fortuna *et al*. 2008, Beltramo *et al*. 2014). In human sperm the effect of kisspeptin on [Ca^2^^+^]_i_ did not occlude the response to stimulation with the CatSper agonist progesterone and was not reduced when applied in the presence of progesterone (Pinto *et al*. 2012). Both KISS1R and kisspeptin itself were detected in the head of human sperm, suggesting that an autocrine action of the peptide may occur. Motility parameters of kisspeptin-treated cells were significantly altered, including an increase in lateral movement of the head and a decrease in linearity of the sperm path, characteristics of hyperactivated sperm (Pinto *et al*. 2012). Ghrelin, another peptide hormone that also acts through mobilisation of stored Ca^2^^+^ (Camina *et al*. 2003), has also been detected in human sperm (Moretti *et al*. 2014). Micromolar concentrations of ghrelin have been shown to increase [Ca^2^^+^]_i_ and motility in rat sperm (Lukaszyk *et al*. 2012), but expression of ghrelin receptors or effect of ghrelin on human sperm [Ca^2^^+^]_i_ have not been investigated.

## Functional significance of Ca^2^^+^-stores

### The acrosome

AR is the fusion between the outer acrosomal membrane and the overlying plasma membrane. Fusion occurs at multiple points, resulting in vesiculation and loss of the fused outer acrosomal membrane/plasmalemma so that the acrosomal content is released and the inner acrosomal membrane becomes the new cell surface. Membrane fusion proteins from the SNARE family are present in the acrosomal region and may be integrated into microdomains that facilitate Ca^2^^+^-regulated membrane fusion in a manner that has been compared with events at presynaptic terminals (De Blas *et al*. 2005, Mayorga *et al*. 2007, Zitranski *et al*. 2010). Zona pellucida proteins interact with sperm surface receptors to activate a signalling cascade leading to AR (Florman *et al*. 2008) and release of acrosomal content at the surface of the zona may, in combination with hyperactivated motility, facilitate zona penetration. However, observation of mouse IVF using sperm with GFP-labelled acrosomes showed that, in addition to cells that undergo AR at the surface of the zona, sperm which arrive having already lost their acrosome (probably within the cumulus) may go on to penetrate the zona and fertilise (Jin *et al*. 2011). Physiological inducers of AR that have been studied (primarily mouse ZP3 and progesterone) induce Ca^2^^+^ influx across the plasma membrane and a sustained rise in [Ca^2^^+^]_i_. O'Toole *et al*. (2000) provided pharmacological evidence that ZP3-induced AR in mouse sperm involved activation of store-operated Ca^2^^+^ influx downstream of Ca^2^^+^ store mobilisation. De Blas *et al*. (2002) showed that in streptolysin-permeabilised human sperm, mobilisation of the acrosomal Ca^2^^+^ store was a requirement for AR even when it was directly induced by introduction of Rab3A into the cytoplasm. Further studies using this permeabilised sperm model have provided information about the mechanisms by which fusion of the plasma and outer acrosomal membranes is regulated, resulting in a detailed model in which mobilisation of the acrosomal store is a central and necessary event (Ruete *et al*. 2014). Stimulation of PLC, leading to generation of IP_3_ and activation of IP_3_Rs in the outer acrosomal membrane may be key to this process (Fukami *et al*. 2001, 2003), but there is also evidence that the acrosomal membrane contains the NAADP-sensitive, Ca^2^^+^-permeable TPC (Calcraft *et al*. 2009) and that NAADP mobilises acrosomal Ca^2^^+^ in mouse sperm (Arndt *et al*. 2014).

### The RNE and calreticulin-containing vesicles

A second area where Ca^2^^+^ storage organelles have been identified in mammalian sperm is at the sperm neck and midpiece (Fig. 1b). Mitochondria have mechanisms for accumulation and release of Ca^2^^+^ (Drago *et al*. 2011, Pizzo *et al*. 2012) and therefore may contribute to Ca^2^^+^ buffering and signalling in this part of the sperm. Inhibition of mitochondrial function in sea urchin sperm, using respiratory inhibitors or uncouplers, causes a rise in [Ca^2^^+^]_i_ and leads to activation of Ca^2^^+^ influx that has characteristics consistent with SOCs (Ardon *et al*. 2009). Treatment with mitochondrial uncouplers (2,4-dinitrophenol, carbonyl cyanide-4-(trifluoromethoxy)-phenyl-hydrazone) also increases [Ca^2^^+^]_i_ in human sperm (J Morris and S Publicover, unpublished observations). Mitochondria may thus contribute to shaping of Ca^2^^+^ signals in sperm. However, the primary stimulus-regulated Ca^2^^+^ storage in this part of the sperm is in the RNE and/or a second, apparently separate group of calreticulin-containing vesicular structures, both of which are sited at the sperm neck region and cytoplasmic droplet (Ho & Suarez 2001, 2003, Naaby-Hansen *et al*. 2001). Mobilisation of Ca^2^^+^ stored in these compartments regulates flagellar activity and treatment of mouse sperm with thimerosal stimulates hyperactivated motility by activating Ca^2^^+^ release from these organelles (Ho & Suarez 2001, Marquez *et al*. 2007). This effect occurs in the absence of extracellular Ca^2^^+^ and can be induced in sperm that are null for CatSper (Marquez *et al*. 2007). In mouse sperm the direction of the major, high-amplitude flagellar bend of hyperactivated sperm can be clearly characterised by reference to the hooked acrosomal cap (pro-hook or anti-hook). Sperm that became hyperactivated during capacitation *in vitro* (due to activation of CatSper) show pro-hook bends whereas those activated by store mobilisation (using thimerosal) show anti-hook bends (Chang & Suarez 2011). When sperm were observed interacting with the lining of isolated mouse oviducts, most hyperactivated cells showed anti-hook bending of the type that is elicited by store mobilisation (Chang & Suarez 2012).

In human sperm a similar effect of store mobilisation is observed. Thimerosal greatly increases the proportion of cells showing hyperactivated motility and 4-aminopyridine, which both alkalinises the cytoplasm (thus activating CatSper) and mobilises stored Ca^2^^+^, is similarly potent (Alasmari *et al*. 2013*a*,*b*). In contrast, manipulations that should activate CatSper (elevation of pH_i_, stimulation with progesterone or prostaglandin E_1_) elevate [Ca^2^^+^]_i_ but have only minor stimulatory effects on the proportion of hyperactivated cells. Instead, these manipulations significantly increase penetration into viscous media (Alasmari *et al*. 2013*a*,*b*, Luo *et al*. 2014).

## Model for interaction of CatSper channels and Ca^2^^+^-stores

Patch clamp recordings have provided no evidence that conventional voltage-operated Ca^2^^+^ channels contribute to Ca^2^^+^ influx in mature mammalian sperm. In mouse sperm null for CatSper1 and the K^+^ channel Slo3, only a small leak current was recorded even at high intracellular pH and strong depolarisation (Zeng *et al*. 2013). CatSper channels in mouse and human sperm are pH- and (weakly) voltage-sensitive, but in human sperm the channel is also ligand-sensitive. Established Ca^2^^+^-mobilising agonists of human sperm such as progesterone and prostaglandin E_1_ have been shown to activate CatSper but also a range of other small molecules including environmental pollutants such as 1,1,1-trichloro-2,2-bis(4-chlorophenyl)ethane, 1,1-bis(4-chlorophenyl)-2,2,2-trichloroethane (4,4′-DDT), *p*,*p*′-dichlorodiphenyldichloroethylene and 4-methylbenzylidene camphor are potent agonists (Tavares *et al*. 2013, Schiffer *et al*. 2014). In addition, agents used to demonstrate cyclic-nucleotide-activated Ca^2^^+^ influx (such as 8-Br-AMP) have been shown directly to activate CatSper by binding at the extracellular surface (Brenker *et al*. 2012). Thus it is possible that a significant proportion of the pharmacological data that apparently support the existence of multiple Ca^2^^+^ influx pathways in sperm are misleading and in fact reflect actions of the drugs on Ca^2^^+^ flux through CatSper channels (Brenker *et al*. 2012). Furthermore, experiments using CatSper null mice provide strong evidence that [Ca^2^^+^]_i_ elevation induced by solubilised ZP is dependent on Ca^2^^+^ influx through the CatSper channel in the flagellum, which then propagates to the head (Xia & Ren 2009; though see Cohen *et al*. (2014)). Interestingly, the ability of solubilised zona to induce AR was not diminished in CatSper-null sperm. These findings not only suggest that CatSper is the primary Ca^2^^+^ influx pathway in mammalian sperm, but also that in human sperm it may act as a Ca^2^^+^-signalling ‘hub’ or ‘node’, such that the effects of diverse agonists are summated/integrated in the rate of Ca^2^^+^ influx into the flagellum (Brenker *et al*. 2012). This is an elegant and simple model for which there is already a significant body of data, but in its basic form it does not address the question of how a sperm can generate and use diverse [Ca^2^^+^]_i_ signals to control diverse Ca^2^^+^-sensitive functions.

**Figure 2:**
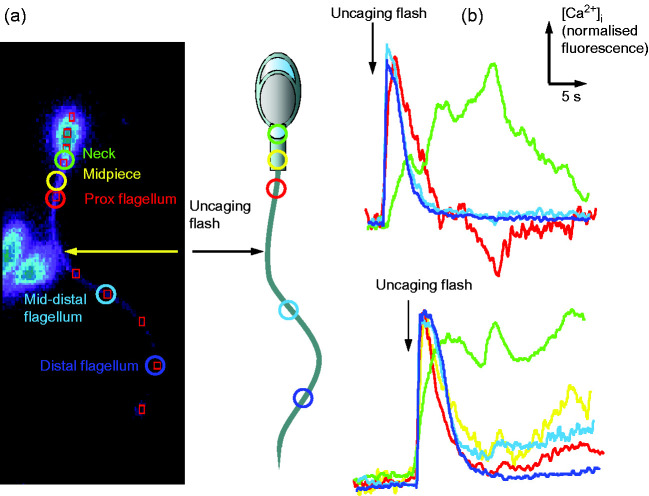
Ca^2^^+^ responses evoked in human sperm by uncaging of Ca^2^^+^ in the flagellum. Cells were labelled with fluo-4 and loaded with caged Ca^2^^+^ (NP-EGTA), then stimulated by an uncaging flash (360 nm laser) at the central flagellum (shown by arrow) while collecting images at 33 Hz. Changes in fluorescence, assessed at each of the positions shown by coloured circles in panel ‘a’, are plotted (normalised to minimum and maximum) in panel ‘b’ using the same colour code. Green, neck; yellow-midpiece; red, proximal flagellum; light blue, mid-distal flagellum; dark blue, distal flagellum.

Mouse sperm null for CatSper are unable to hyperactivate (Carlson *et al*. 2003) and evidence from clinical cases suggests that CatSper is also required for normal levels of motility in human sperm (Avenarius *et al*. 2009, Smith *et al*. 2013). Why, then, is manipulation of Ca^2^^+^ stores more effective in inducing hyperactivated motility than treatments targeted to CatSper (Alasmari *et al*. 2013*b*)? We have proposed that CatSper activation acts as a trigger and consequent elevation of flagellar [Ca^2^^+^]_i_ stimulates secondary release of stored Ca^2^^+^ at the sperm neck, either by stimulating synthesis of IP_3_ or by CICR, leading to hyperactivation (Alasmari *et al*. 2013*b*). Mathematical modelling of the Ca^2^^+^ signals induced by CatSper activation in mouse sperm suggests that diffusion of Ca^2^^+^ from the flagellum cannot explain the [Ca^2^^+^]_i_ increase that occurs at the sperm head upon activation of CatSper and that such a secondary Ca^2^^+^ release at the neck region must occut (Olson *et al*. 2010, 2011, Li *et al*. 2014). Recently we have investigated the occurrence of such secondary responses in human sperm by uncaging Ca^2^^+^ in the principal piece of the flagellum. Uncaging induces a clear [Ca^2^^+^]_i_ transient in the flagellum that decays within 5–10 s. At the neck region of the sperm the transient is truncated and rises more slowly, consistent with diffusion of Ca^2^^+^ from the uncaged pool, but in a small proportion of cells (∼10%) we have observed a late [Ca^2^^+^]_i_ response at the neck region, often including multiple peaks (Fig. 2). The low incidence of this secondary Ca^2^^+^-mobilisation is consistent with our observation that, though direct release of stored Ca^2^^+^ can induce hyperactivated motility in the majority of human sperm, only a small proportion of cells hyperactivate upon activation of CatSper (Alasmari *et al*. 2013*a*,*b*).

**Figure 3:**
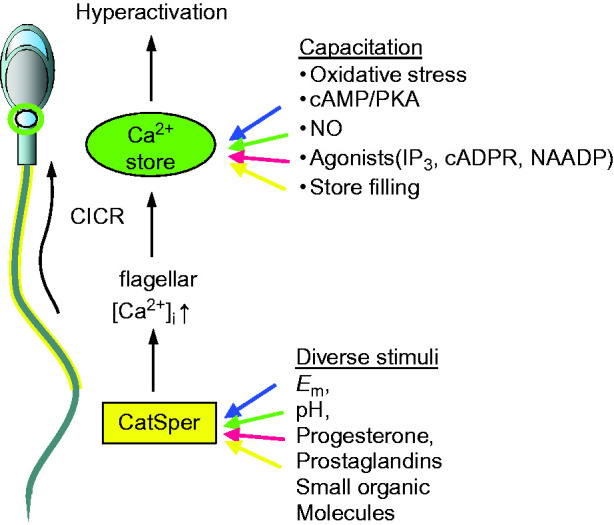
Model for triggering/regulation of CatSper-activated hyperactivation. CatSper channels in the flagellum (yellow box; shown by yellow shading on sperm flagellum) are activated by diverse stimuli including intracellular pH (pH_i_), membrane potential (*E*_m_), progesterone, prostaglandins and other organic molecules. Ca^2^^+^ from the flagellum diffuses forward, raising [Ca^2^^+^]_i_ at the sperm neck and can mobilise stored Ca^2^^+^ by Ca^2^^+^-induced Ca^2^^+^ release (CICR). Susceptibility of the store to CICR is potentially regulated/sensitised by processes occurring during capacitation, including cAMP signalling, oxidative stress and S-nitrosylation as well as Ca^2^^+^ store filling and effects of agonists on Ca^2^^+^-store release channels.

Ca^2^^+^-store-mediated [Ca^2^^+^]_i_ oscillations occur more readily in sperm incubated for a prolonged period (>24 h) under capacitating conditions (Kirkman-Brown *et al*. 2004). Capacitation involves generation of ROS and RNS (Herrero *et al*. 1999, 2001, Aitken & Nixon 2013) and we have observed that store mobilisation is sensitised and induced by low concentrations of NO donors, through a mechanism that involves protein S-nitrosylation (Machado-Oliveira *et al*. 2008). RyRs were detected in the human sperm nitrosoproteome (Lefievre *et al*. 2007) and it is well-established that IP_3_Rs and RyRs are sensitised by oxidative stress (Bootman *et al*. 1992, Sayers *et al*. 1993, Stoyanovsky *et al*. 1997, Meissner 2004, Bansaghi *et al*. 2014) (see ‘Ca^2^^+^ stores and their regulation’). We propose that CICR from the sperm neck Ca^2^^+^-store is regulated during capacitation, perhaps through the effects of oxidative stress on Ca^2^^+^ release channels (Alasmari *et al*. 2013*b*) (Fig. 3).

## Final remarks

The central role of [Ca^2^^+^]_i_ signalling in the physiology of mammalian sperm and the pivotal importance of CatSper in this process are well established – mice null for CatSper are infertile (Ren *et al*. 2001) and in men CatSper lesions are associated with impaired sperm function (Avidan *et al*. 2003, Avenarius *et al*. 2009, Zhang *et al*. 2009, Smith *et al*. 2013). The available evidence suggests that Ca^2^^+^-stores also play important roles in both AR and the regulation of motility. Future studies should address the mechanisms by which store mobilisation is achieved (both by CICR and by agonist-induced generation of Ca^2^^+^-mobilising 2nd messengers) and regulated, particularly the significance of capacitation in Ca^2^^+^-store filling and in sensitising Ca^2^^+^ release mechanisms. Also, similarly to the important species differences in expression and function of sperm ion channels between human and mouse sperm (Brenker *et al*. 2014, Miller *et al*. 2015), there may be differences in store-regulation and/or function between species. An intriguing possibility is that, at least in human sperm, it may prove possible to bypass the effects on motility of lesions in the expression, function or regulation of CatSper channels by pharmacological activation of stored Ca^2^^+^ release.
